# *CsWRKY17* enhances Al accumulation by promoting pectin deesterification in tea plant

**DOI:** 10.1093/hr/uhaf085

**Published:** 2025-03-11

**Authors:** Danjuan Huang, Jianqiang Ma, Xun Chen, Hongjuan Wang, Rongrong Tan, Long Jiao, Jiedan Chen, Yingxin Mao, Liang Chen

**Affiliations:** Key Laboratory of Tea Resources Comprehensive Utilization of Ministry of Agriculture and Rural Affairs, Fruit and Tea Research Institute, Hubei Academy of Agricultural Sciences, 10 Nanhu Avenue, Hongshan District, Wuhan 430064, China; Key Laboratory of Biology, Genetics and Breeding of Special Economic Animals and Plants, Ministry of Agriculture and Rural Affairs, Tea Research Institute of the Chinese Academy of Agricultural Sciences, 9 South Meiling Road, Xihu District, Hangzhou 310008, China; Key Laboratory of Biology, Genetics and Breeding of Special Economic Animals and Plants, Ministry of Agriculture and Rural Affairs, Tea Research Institute of the Chinese Academy of Agricultural Sciences, 9 South Meiling Road, Xihu District, Hangzhou 310008, China; Key Laboratory of Tea Resources Comprehensive Utilization of Ministry of Agriculture and Rural Affairs, Fruit and Tea Research Institute, Hubei Academy of Agricultural Sciences, 10 Nanhu Avenue, Hongshan District, Wuhan 430064, China; Key Laboratory of Tea Resources Comprehensive Utilization of Ministry of Agriculture and Rural Affairs, Fruit and Tea Research Institute, Hubei Academy of Agricultural Sciences, 10 Nanhu Avenue, Hongshan District, Wuhan 430064, China; Key Laboratory of Tea Resources Comprehensive Utilization of Ministry of Agriculture and Rural Affairs, Fruit and Tea Research Institute, Hubei Academy of Agricultural Sciences, 10 Nanhu Avenue, Hongshan District, Wuhan 430064, China; Key Laboratory of Tea Resources Comprehensive Utilization of Ministry of Agriculture and Rural Affairs, Fruit and Tea Research Institute, Hubei Academy of Agricultural Sciences, 10 Nanhu Avenue, Hongshan District, Wuhan 430064, China; Key Laboratory of Biology, Genetics and Breeding of Special Economic Animals and Plants, Ministry of Agriculture and Rural Affairs, Tea Research Institute of the Chinese Academy of Agricultural Sciences, 9 South Meiling Road, Xihu District, Hangzhou 310008, China; Key Laboratory of Tea Resources Comprehensive Utilization of Ministry of Agriculture and Rural Affairs, Fruit and Tea Research Institute, Hubei Academy of Agricultural Sciences, 10 Nanhu Avenue, Hongshan District, Wuhan 430064, China; Key Laboratory of Biology, Genetics and Breeding of Special Economic Animals and Plants, Ministry of Agriculture and Rural Affairs, Tea Research Institute of the Chinese Academy of Agricultural Sciences, 9 South Meiling Road, Xihu District, Hangzhou 310008, China; Yunnan Key Laboratory of Tea Germplasm Conservation and Utilization in the Lancang River Basin, West Yunnan University, 2 Xuefu Road, Linxiang District, Lincang 677000, China

## Abstract

The tea plant (*Camellia sinensis*) is a typical crop that accumulates aluminum (Al). Although the physiological mechanisms by which this occurs are well understood, their molecular mechanisms remain elusive. Here, an integrative approach combining quantitative trait locus (QTL) mapping of controlled hybridized populations and comparative transcriptomic analysis using samples treated with different Al concentrations was applied to identify candidate genes associated with Al accumulation in tea plants. Consequently, 41 candidate genes were selected using genome functional annotation of the *qAl09* locus in the region of 35 256 594–57 378 817 bp on chromosome 7. Finally, a key gene, *CsWRKY17*, was identified as encoding a nucleus-localized transcription factor involved in regulating Al accumulation in tea plants, given the finding of a high correlation between its expression level and Al content in leaves. Overexpression of *CsWRKY17* in *Arabidopsis* increased pectin deesterification, sensitivity to Al stress, and Al accumulation in leaves. Expression of the pectin methylesterase gene *CsPME6* was found to be highly consistent with *CsWRKY17* expression under various Al concentrations. In addition, experiments using a yeast monoclonal, electrophoresis mobility shift assay and dual-luciferase reporter (DLR) system confirmed that *CsWRKY17* activated *CsPME6* promoter activity. Antisense oligodeoxynucleotide silencing revealed a positive association between *CsPME6* expression and Al accumulation in tea shoots. In conclusion, this study suggests that *CsWRKY17* promoted the process of pectin deesterification by binding to the *CsPME6* promoter, thereby enhancing Al enrichment in tea plants. Our findings lay the foundation for studying the precise mechanisms through which Al enriched in tea leaves.

## Introduction

Tea is one of the most widely consumed beverages worldwide. The tea plant (*Camellia sinensis*) is recognized as a poly-aluminum species because of its capacity to absorb and store high concentrations of aluminum (Al), which also enhances its growth at optimal levels [1–3]. After being absorbed by roots, Al is transported upward through the xylem in the form of complexes via its chelation with organic acids, catechins, phosphoric acid, or fluorine and then stored in leaves [4–6]. Within leaves, the Al concentration increased with maturation of leaves [7, 8]. Excessive intake of Al has adverse effects on the human body, such as memory loss, growth retardation, and osteoporosis [9]. Typically, the drinking of tea at normal levels will not cause excessive Al intake [10]. However, by processing mature leaves of tea plants into matcha, instant tea, tea concentrate and tea-containing food realizes comprehensive utilization of tea processing, substantial economic value is added, but this also increases the risk of direct Al intake. To ensure the sustainable and healthy development of the tea industry, it is thus of great significance to reduce the above-mentioned potential safety risks through the selection and utilization of tea varieties that are less enriched in Al.

Studies have shown that tea plants’high tolerance and enrichment of Al are due to efficient Al chelation and segregation in the cell walls of their leaves [11]. Gao *et al.* [12] found that 69.8% and 75.2% of Al in tea plant roots and leaves, respectively, were stored in the cell wall. Confocal laser scanning microscopy further confirmed these findings, showing a higher Al fluorescence signal in the cell wall than in other cell components [13]. This form of storage effectively protects the cytoplasm of tea plants against Al-associated toxicity. However, the role of cell walls in the enrichment of Al and the underlying physiological and molecular mechanisms in tea plants require further exploration.

The cell wall, which primarily consists of pectin, hemicellulose, and cellulose, acts as the first barrier that prevents heavy metals from entering into cell protoplasts [14, 15]. Pectin, which is expected to be the primary binding site for metal ions in the cell wall because of its abundance of carboxyl groups, acquires a negative charge upon dissociation [16]. Once pectin is synthesized in the Golgi body and transported to the cell wall through vesicles in a highly esterified form, it undergoes deesterification by pectin methylesterase (PME). This process removes the methylester group (–COOCH_3_) from the pectin side chain, forming carboxylic groups (–COO) that bind cations [17, 18]. Therefore, to reduce Al enrichment in plant cell walls, it is crucial to increase the degree of pectin esterification. PME, a member of the carbohydrate esterase family 8, modifies pectin structure, thereby regulating intercellular viscosity, rhizosphere pH, and cell wall ion-binding capacity. This regulation is vital for plant growth, development, and environmental stress response [19]. However, it has been shown that only a few PMEs contribute to the accumulation of Al in plant tissues. For example, it was found that 8 out of 35 *OsPMEs* in rice were upregulated under Al stress; overexpression of *OsPME14* enhanced the PME activity and Al content in root tips, thus leading to decreased Al resistance in rice [20]. Moreover, in *Arabidopsis*, *PME46* was found to increase Al tolerance by lowering PME activity, facilitating methylated pectin accumulation, and consequently decreasing the amount of Al bound to the cell wall [21]. In another study, Li *et al.* [22] employed immunofluorescence and specific monoclonal antibodies LM19 and LM20 to measure the degree of pectin methylesterification in the root tips of tea plants treated with various Al concentrations. As the Al concentration increased, pectin demethylesterification of root tips increased along with the expression of *CsPME2, CsPME7*, and *CsPME21*. These results indicate that methyl esterification plays a vital role in Al binding to the cell walls of tea plants.

**Figure 1:**
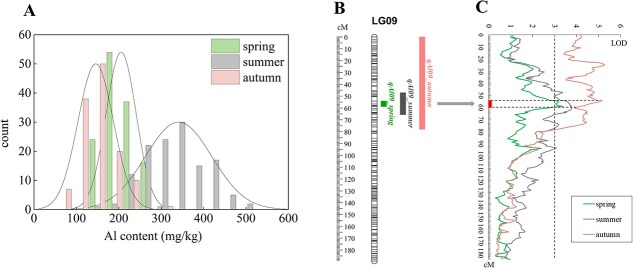
QTL analysis of Al content in the ‘YS’ × ‘BD’ F_1_ population. (A) Frequency distribution of F_1_ Al content in different seasons; (B) Distribution of Al-related QTLs on LG09; (C) LOD score curve of QTLs for Al content on LG09.

Transcriptomic analysis has identified genes related to Al uptake and accumulation in tea plants [23–25]. However, most of this work was based on omics technology using reverse genetics. Given the lack of a system for the stable genetic transformation of tea plants, identifying key genes among a large number of candidate genes remains an important bottleneck restricting related research. In this study, a combination of linkage and comparative transcriptomic analyses was used to genetically analyze QTLs associated with Al content in the shoots of tea plants in spring, summer, and autumn. Then, the candidate gene was screened and its function was preliminarily analyzed. Our research sheds light on the molecular mechanisms by which Al is enriched in tea leaves and provides theoretical guidance and new genetic resources for improving varieties of tea plants with low Al content.

## Results

### Variation and QTL analysis of Al accumulation

Descriptive statistics of Al content in the parents and F_1_ population across the three seasons are presented in Table S1. A *t*-test analysis revealed that ‘Yingshuang’ (YS) consistently exhibited significantly higher Al content than ‘Beiyao Danzhu’ (BD) across all three seasons. The mean Al contents for both parents and progeny were in the following order: summer > spring > autumn. This may be because the tea plants have already undergone two deep pruning when autumn tea samples were picked. The Al content within the F_1_ population varied seasonally, ranging from 119.80 to 317.60 mg/kg in spring, 165.40 to 624 mg/kg in summer, and 63.50 to 296.8 mg/kg in autumn. The population coefficient of variation (CV) for Al accumulation was significantly higher in autumn than in spring. Al accumulation in the F_1_ population was considered to be a quantitative trait for exhibiting a normal distribution model with transgressive segregation (Fig. 1A).

Six QTLs were found to be scattered among four linkage groups (LG): LG01, LG03, LG09, and LG12 (Table S2). LG12 contained the highest number of QTLs (3), while the remaining linkage groups each harbored one. *qAl01*, *qAl03*, *qAl12-1*, and *qAl12-3* were detected exclusively in spring. One QTL was detected in at least two seasons, and another in all three. *qAl12-2* was consistently detected in spring and summer, spanning 175.395–177.318 cM, with a maximum logarithm of odds ratio (LOD) score of 3.96. Similarly, *qAl09* was stably detected across spring, summer, and autumn (Fig. 1B, C), within a range of 54.17–58.852 cM and a maximum LOD score of 5.29. The phenotypic variance explained of all tested QTLs varied between 10.7% and 17.6%.

### Changes in Al content in leaves and cell walls at different Al concentrations

**Figure 2:**
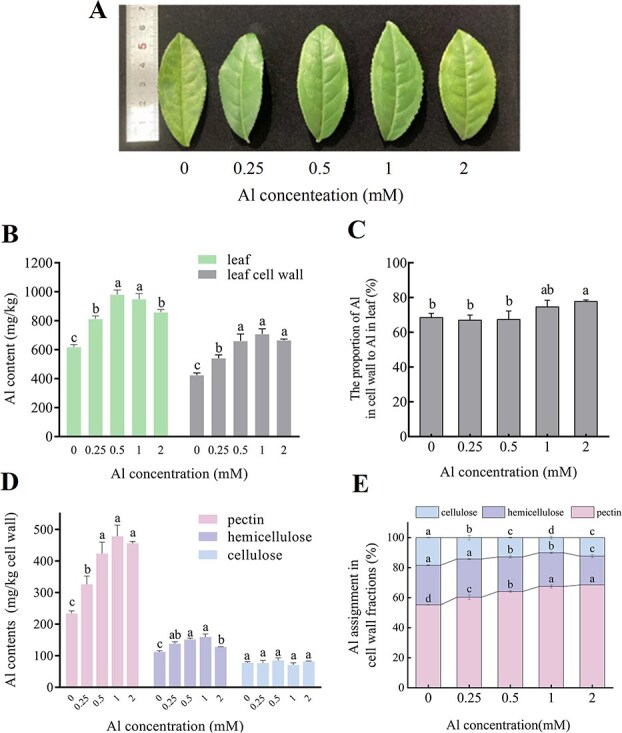
Effects of different Al concentrations on Al accumulation in tea plant leaves and leaf cell walls. (A) Leaf phenotype changes. (B) Al contents in leaves and leaf cell walls. (C) The proportions of Al in cell walls to Al in leaves (%). (D) The Al contents in pectin, hemicellulose and cellulose. (E) Percentages of Al in pectin, hemicellulose and cellulose relative to total cell wall Al content.

One-year-old ‘E’cha 1’ cuttings were subjected to five Al concentrations (0, 0.25, 0.5, 1, and 2 mM) for 30 days. The third leaf from the new shoot was collected for analysis. Fig. 2A shows that regreening of tea leaves occurred in the absence and presence of 2 mM Al. Upon treatment, with increasing concentrations of Al, the leaf and leaf cell wall Al content exhibited a trend of rising and then decreasing (Fig. 2B). The Al content cell wall/leaf percentage ranged from 66.93% to 77.71%, indicating that the cell wall is the main site at which Al is stored in the leaves of tea plants (Fig. 2C). As shown in Fig. 2D, the Al content in the three cell wall fractions (pectin, cellulose, and hemicellulose) significantly increased under exogenous Al treatment compared with that in the absence of Al. Among these, the pectin Al content exhibited notable increases of 39%, 81%, 101%, and 94% at Al concentrations of 0.25, 0.5, 1, and 2 mM, respectively, compared with that at 0 mM. Al was predominantly enriched in pectin, accounting for 55.21%–68.52% of the total Al content in the cell wall, followed by hemicellulose (19.19%–26.41%) and then cellulose (10.05%–18.38%). The proportion of pectin Al increased with increasing Al concentration, whereas the proportions of Al in hemicellulose and cellulose showed decreasing trends (Fig. 2E). These findings suggest that the accumulation of Al in pectin is a critical mechanism by which Al is enriched in tea plants.

The results illustrate that, in the absence of Al, little new root formation occurred in tea plants, and the old roots became brown (Fig. S1A). Conversely, low Al concentrations (0.25–1 mM) significantly promoted new root growth, whereas high Al concentration (2 mM) produced only a limited number of new roots, confirming Al’s essential role in new root development. The Al content in the roots and root cell walls increased dramatically with increasing Al concentration (Fig. S1B). Compared with the findings in the absence of Al, the proportions of Al in the cell wall relative to root were not significantly affected by treatment with 0.25 mM Al. However, a significant increasing trend in this proportion was observed for 0.5 and 1 mM Al (Fig. S1C). Analysis of Al content and its distribution across the three root cell wall components revealed that Al was primarily localized in pectin, which accounted for 53.93%–64.4% of the total Al in root cell walls (Fig. S1D and E).

**Figure 3:**
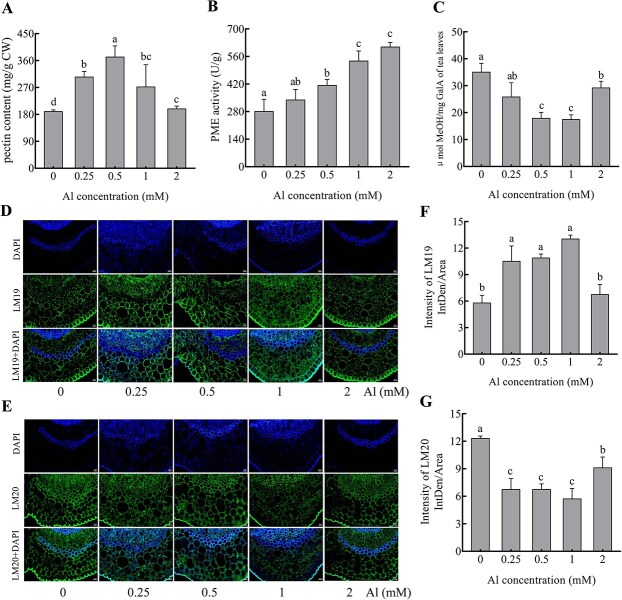
Influence of treatments with different Al concentrations on the pectin content, PME activities and structure of the cell wall of tea plant leaves. (A) Total pectin content. (B) PME activities. (C) Degree of HG DM. (D, E) Images of HGs labeled by LM19 and LM20. The first row indicates DAPI (4′,6-diamidino-2-phenylindole) staining, the second row indicates LM19 or LM20 epitope staining, and the third row indicates the superposed images of DAPI and LM19 or LM20 immunolocalization for epitope staining. The scale bar in in the bottom right represents 20 μm. (F) The fluorescence intensity of LM19-labeled HG structures. (G) The fluorescence intensity of LM20-labeled HG structures. LM19 and LM20 marked de-esterified pectin and esterified pectin, respectively.

Fourier transform infrared spectroscopy (FT-IR) can be used to observe changes in a compound’s functional groups at different wavelengths. In a previous study, two peaks at 1749 and 1630 cm^−1^ corresponding to esterified and nonesterified carboxyl groups of pectin were identified by infrared spectroscopy of peach cell walls [26]. Therefore, these two wavelengths can serve as a reference for indicating the esterification of pectin in tea plants under varying Al concentrations. Upon FT-IR analysis, the wave trend of the FT-IR spectrum of leaf and root cell walls remained consistent across treatments with different Al concentrations, although peak intensities varied notably (Fig. S2). By using the intensity of the specific absorption peak of the C–H bond (I_2910_ + I_2843_) as a reference, the ratio of peak intensity to the reference peak intensity was calculated. This ratio represents the relative content, enabling semiquantification of –COOH, –COOCH_3_, and –COO– [27, 28]. In tea plant leaves, a peak at 1733 cm^−1^ corresponds to the nonionized carboxyl functional group of cell wall pectin. With increasing Al concentration, the intensity of this peak displayed a trend of initially decreasing and then increasing within the concentration range of 0– 2 mM. In contrast, a peak at 1640 cm^−1^ represents the carboxyl functional group in the ionized state, which showed the opposite trend of first increasing and then decreasing in peak intensity (Table S3). This indicates that, compared with the findings in the absence of Al, the amount of ionized carboxyl functional groups in leaf cell wall pectin significantly increased after Al treatment. Meanwhile, the total amount of carboxyl functional groups tended to first show an increase, and then a decrease (Table S3), which was consistent with the change in leaf pectin content (Fig. 3A). In the FT-IR analysis of roots, although the amount of carboxyl functional groups in the ionized state of pectin did not differ significantly among the treatments with 0, 0.25, and 0.5 mM Al, its level was higher under Al treatment than in the absence of Al (Table S3). The pectin content in roots increased with increasing Al concentrations in the range of 0–0.5 mM but then plateaued, showing no significant difference among concentrations of 0.5, 1, and 2 mM (Fig. S3A). Furthermore, PME activities displayed an upward trend with the increasing Al concentration in both leaves and roots (Fig. 3B; Fig. S3B), implying that PME-catalyzed pectin demethylesterification is strongly correlated with Al binding to the cell wall of tea plants.

It was previously reported that homopolygalacturonic acid (HG), the predominant pectin polysaccharide, undergoes demethylesterification catalyzed by pectin methylesterase , revealing carboxyl groups [29]. The degree of methylesterified (DM) HG correlates with Al enrichment by pectin. We then determined the HG DM using the ratio of methanol production to galacturonic acid content. In leaves, DM values decreased with increasing Al concentration from 0 to 1 mM but then increased at 2 mM (Fig. 3C). We also used the antibodies LM19 and LM20 for fully de-esterified and fully esterified pectin, respectively, to identify the HG structure of cell wall pectin via immunohistochemical staining, with the mean fluorescence intensity calculated via ImageJ software. The results of immunohistochemical staining indicated that the epitope signals of LM19 were significantly enhanced under treatment with 0.25–1 mM Al, but decreased at 2 mM (Fig. 3E). However, the mean fluorescence intensity surpassed that of the treatment without Al, suggesting Al’s role in promoting the formation of completely de-esterified pectin in tea leaf cell walls. The LM20 antibody signal diminished across all four Al concentrations (Fig. 3F), implying that Al facilitates the conversion of high-ester pectin to low-ester pectin in tea leaf cell walls. Interestingly, at an Al concentration of 2 mM, the intensity of the LM19 antibody signal weakened, while that of the LM20 signal increased, possibly due to physiological stress on the tea plants induced by high Al, disrupting pectin metabolism in the cell wall. In the roots, DM values showed a consistent decreasing trend with increasing Al concentration (Fig. S3C). This pattern aligned with changes in the strength of the LM20 signal (Fig. S3G), but it contrasted with the trend observed for LM19 (Fig. S3F). Additionally, the LM19 signal was significantly stronger in epidermal tissue than in the cortex (Fig. S3D), whereas the LM20 signal was stronger in the cortex, endodermis, and central cells than in the epidermis (Fig. S3E). These findings indicate that epidermal tissue contained higher levels of completely de-esterified pectin, whereas esterified pectin was primarily localized in the cortex, endodermis, and central cells.

Hemicellulose in *Arabidopsis* has been found to play an even more important role than pectin in binding Al [30]. To investigate this, we examined the effects of different Al concentrations on the hemicellulose content. The results showed that hemicellulose content in leaves increased under Al treatment, peaking at moderate concentrations before declining as the Al concentration increased further (Fig. S4A). In contrast, the hemicellulose content in roots exhibited a consistent upward trend with increasing Al concentration (Fig. S4B). We also analyzed the correlations between pectin, hemicellulose, PME activity, DM, and cell wall Al content in tea plants. The contents of hemicellulose (*r* = 0.78, *P* = 0.0077) and PME activity (*r* = 0.71, *P* = 0.049) positively correlated with cell wall Al content in leaves. Similarly, pectin (*r* = 0.90, *P* < 0.0001), hemicellulose (*r* = 0.81, *P* = 0.0003), and PME activity (*r* = 0.76, *P* = 0.0001) were significantly correlated with cell wall Al content in roots. In contrast, DM exhibited a significant negative correlation with cell wall Al content (*r* = −0.77, *P* = 0.0087, in leaves and *r* = −0.923, *P* < 0.0001, in roots, respectively). These findings imply that Al-induced increases in pectin, hemicellulose, and PME activity, along with Al-induced decrease in DM, play a crucial role in Al enrichment in tea plants.

### Candidate gene screening by combining QTL results with transcriptomic analysis

**Figure 4:**
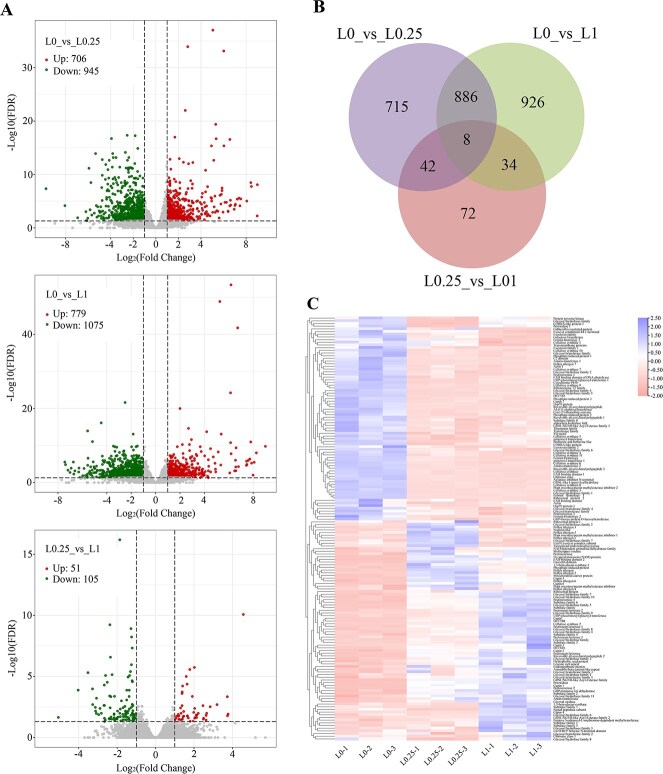
Analysis of DEGs under different Al concentrations. L0, L0.25, and L1 represent leaves treated with 0, 0.25, and 1 mM Al, respectively. (A) Volcano maps of L0 vs. L0.25, L0 vs. L1 and L0.25 vs. L1 DEGs. (B) Venn diagram of DEGs. (C) Cluster analysis of DEGs involved in cell wall metabolism induced by Al treatment.

The transcriptomic data of third leaves treated with 0 (L0), 0.25 (L0.25), and 1 (L1) mM Al were evaluated, and gene sequence comparison was conducted using the ‘Shuchazao’ genome as a reference. More than 86% of the reads from L0, L0.25, and L1 effectively matched the reference sequence (Table S4). Upon applying the thresholds of false discovery rate <0.001 and |log2| value ≥1, 1651 and 1854 differentially expressed genes (DEGs) were identified in L0.25 and L1, respectively, using L0 for comparison (Fig. 4A). However, only 156 DEGs were found in the L0.25 vs. L1 comparison (Fig. 4A). The Venn diagram showed that 894 DEGs were shared between the comparisons of L0 vs. L0.25 and L0 vs. L1, which was significantly more than the 50 and 42 DEGs shared between the comparisons of L0 vs. L0.25 and L0.25 vs. L1, and between L0 vs. L1 and L0.25 vs. L1, respectively (Fig. 4B). Hierarchical cluster analysis revealed that L0.25 and L1 grouped into a single class, while L0 formed a separate class (Fig. S5). Taking these findings together, the absence of Al resulted in greater changes in leaf gene expression, affecting the normal growth of the tea plants more than various Al concentrations within the suitable growth range.

Given the invaluable finding that Al in tea leaves is primarily concentrated in the cell wall, we further analyzed functional genes related to cell wall metabolism (Fig. 4C). We identified 158 DEGs, including those correlated with glycosyl hydrolase (27), cellulose synthase (12), glycosyl transferase (5), PME (5), pectin acetylesterase (4), PME inhibitor (3), β-1,3-glucan synthase (2), and xyloglucan endotransglycosylase. Among the 27 glycosyl hydrolases, 12 were upregulated in response to Al treatment. Additionally, we identified a PME gene (CSS0014883) whose expression was upregulated by 76-fold and 99-fold under 0.25 and 1 mM Al treatments, respectively.

Based on QTL mapping of the F_1_ genetic population from the YS × BD cross, loci were identified at 54.17–58.83 cM in LG09 (corresponding to Chr07) stably across spring, summer, and autumn. Using tag sequences in the Tea plant Genome Variation Database (TeaGVD) (http://www.teaplant.top/teagvd) [31], this interval was mapped to 35 256 594–57 378 817 bp on chromosome 7, covering ~22.1 Mb and encompassing 545 genes. GO, KEGG, and Pfam annotations, combined with previous reports [32–34], helped identify 41 candidate genes within this region (Table S5). Five genes with significant positive correlations with Al content were selected. Genomic annotations identified that these genes encoded terpene synthase, WRKY transcription factor, and zinc finger CCCH domain-containing proteins. Expression of the gene encoding the WRKY transcription factor exhibited correlation coefficients of 0.931 and 0.946 with Al content in leaves and the leaf cell wall, respectively. This transcription factor named *CsWRKY17* by Zhao *et al.* [35] was thus chosen for subsequent functional analysis.

### Transgenic functional analysis of *CsWRKY17*

The full-length of *CsWRKY17* was cloned from ‘E’cha 1’. The complete coding sequence (CDS) of *CsWRKY17* was 1407 bp, encoding 468 amino acids (Table S6), for which the domain pattern analysis indicated two conserved WRKY domains (WRKYGQK) and one C2H2-type zinc finger structure (Fig. S6A). It was classified into subfamily I of the WRKY transcription factor family. Phylogenetic analysis with homologous proteins from *Arabidopsis thaliana*, rice, kiwi, and grape showed that CsWRKY17 is most closely related to AcWRKY20 of *Actinidia chinensis*, with 64.37% homology (Fig. S6B). Meanwhile, Cell-PLoc 2.0 (http://www.csbio.sjtu.edu.cn/bioinf/Cell-PLoc-2/) prediction indicated nuclear localization of *CsWRKY17*. To confirm this, the *CsWRKY17* gene fusion expression vector and the empty vector PC1300S-GFP were cotransformed into *Arabidopsis* protoplasts using a nuclear localization marker (Fig. S6C). Consequently, the resulting green fluorescence from the recombinant CsWRKY17 and GFP proteins, along with the red fluorescence from the nuclear labeling dye, confirmed nuclear colocalization.

**Figure 5:**
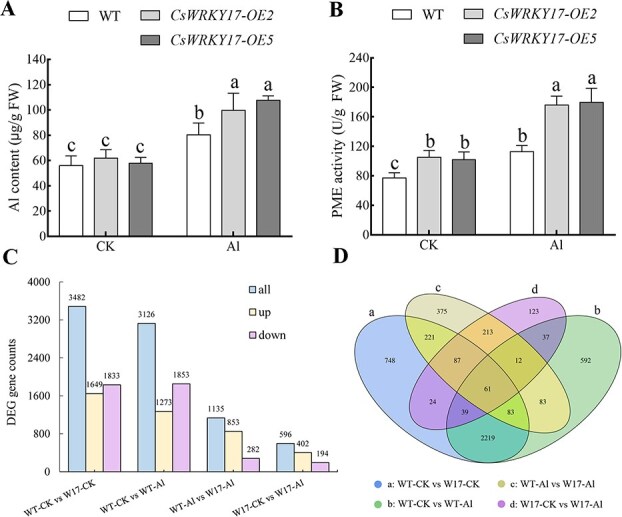
Effect of Al on the leaves of WT and *CsWRKY17-*overexpressing *Arabidopsis* (W17). (A) Al content in *Arabidopsis* leaves. (B) PME activities in *Arabidopsis* leaves. (C) DEG numbers in WT and *CsWRKY17-*overexpressing plants. (D) Venn plot of WT and *CsWRKY17-*overexpressing plants.

To identify the function of *CsWRKY17*, a vector for overexpression *CsWRKY17* was constructed, and *A. thaliana* was transformed by infecting flower buds with *Agrobacterium*. Two T3 generation homozygous transgenic lines, *CsWRKY17*-OE2 and *CsWRKY17*-OE5, were screened for overexpression (Fig. S7). We then conducted a 24-well plate transfer treatment and observed that the Al content did not differ significantly between wild-type (WT) and *CsWRKY17*-OE-overexpressing plants in the control group. However, upon treatment with Al, the leaf Al content and PME activities in *A. thaliana* of *CsWRKY17*-OE plants were higher than those in WT (Fig. 5A and B).

To investigate the enhanced Al tolerance in *A. thaliana* transgenic plants expressing *CsWRKY17*, we performed RNA-seq analysis on leaves from both WT and *CsWRKY17*-overexpressing *A. thaliana* plants subjected to Al treatment. RNA-seq data showed 3126 and 596 DEGs in WT and *CsWRKY17-*overexpressing plants under Al treatment, representing a 5.24-fold difference. The upregulated genes numbered 1273 and 402, respectively, involving a 3.16-fold difference, indicating greater sensitivity to Al treatment in the WT (Fig. 5C). Comparing DEGs between WT and *CsWRKY17-*overexpressing plants, 452 DEGs overlapped between the control and Al treatment (Fig. 5D). In the control group, there were 3030 unique DEGs, whereas under Al treatment, there were 683. This indicates that the differences in gene expression between the two groups were more pronounced in the absence of Al than in its presence. KEGG enrichment analysis of the DEGs indicated significant enrichment of the MAPK signaling pathway in both WT and *CsWRKY17*-overexpressing *Arabidopsis* plants under Al treatment (Fig. S8). However, WT plants exhibited additional significant enrichment in metabolic pathways, such as oxidative phosphorylation, phagosomes, and carbon fixation in photosynthetic organisms. In contrast, overexpressed plants exhibited enrichment in the circadian rhythm, hormone signal transduction, flavonoid biosynthesis, and anthocyanin biosynthesis. Analysis of the W17-CK vs. W17-Al group comparison revealed 20 cell wall-related genes, including 4 pectin methylase genes (3 upregulated, 1 downregulated), whereas the WT-CK vs. WT-Al group comparison revealed 95 cell wall-related genes, including 9 pectin methylase genes (1 upregulated, 8 downregulated).

### *CsWRKY17* targets and regulates the *CsPME6* promoter

**Figure 6:**
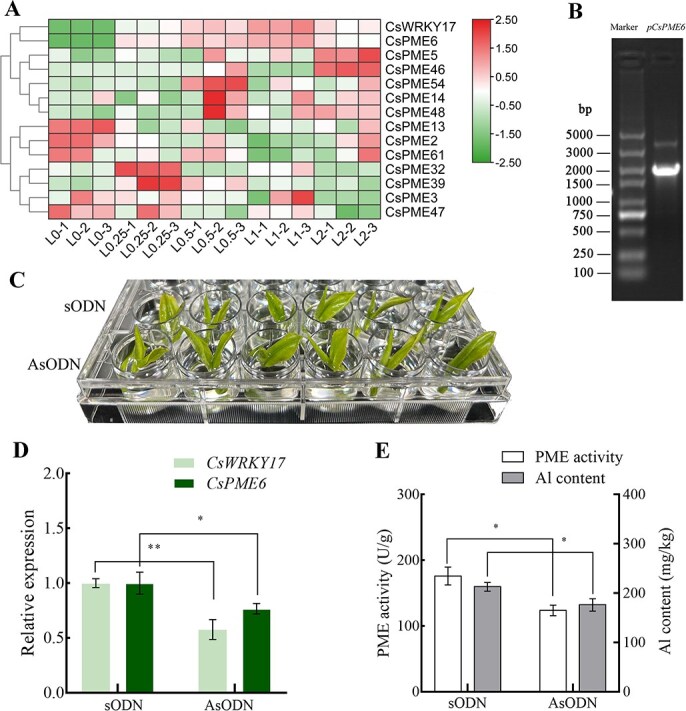
The gene silencing experiment of *CsWRKY17* in tea plant leaves. (A) Expression patterns of *CsPMEs* and *CsWRKY17* under different Al concentrations. (B) *CsPME6* promoter cloning. (C) Tea shoots were immersed with 20 μM asODN for 48 h, with sODN serving as a control. (D) The expression levels of *CsWRKY17* and *CsPME6.* (E) Changes in PME activity and cell wall Al content. Asterisks denote a significant difference (^*^P < 0.05; ^**^P < 0.01).

Based on the hidden Markov model of the PME domain (PF01095), 66 pectin methylase genes (*CsPMEs*) were identified in the ‘Shuchazao’ genome [36]. The analysis of cis-acting elements in the promoters of these 66 *CsPMEs* (2 kb upstream of the 5′ terminal) revealed that 37 *CsPMEs* contained WRKY transcription factor-specific binding W-box elements. The relative expression of these 37 genes in the third tea leaves was detected using quantitative real time polymerase chain reaction (qRT-PCR) under five Al concentrations (0, 0.25, 0.5, 1, and 2 mM for 30 days). Among these, 13 *CsPME* genes responded to various Al concentrations, with *CsPME6* (CSS0014883) showing significant upregulation under all Al concentrations compared with the control (Fig. 6A). This result aligns with our previous transcriptomic findings, in which *CsPME6* expression increased 76- and 99-fold under treatments with 0.25 and 1 mM Al, respectively. These findings were also consistent with the expression pattern of *CsWRKY17* (Fig. 6A) and the trend of changes in Al content in the leaf cell wall (Fig. 2B). We then examined the expression of *CsWRKY17* and *CsPME6* in different tissues using qRT-PCR. The results revealed that *CsWRKY17* was more highly expressed in old leaves and fruits (Fig. S9A), whereas *CsPME6* showed high expression in stems, young leaves, and mature leaves (Fig. S9B).

We cloned the promoters of *CsPME6* (Fig. 6B) and identified a specific W-box element in pro-CsPME6 sequences (Table S7). First, we examined the impact of *CsWRKY17* on *CsPME6* expression, PME activity, and cell wall Al content using antisense oligonucleotide (asODN) technology to transiently knock down *CsWRKY17* expression in tea plants (Fig. 6C). In contrast to the findings in the control (sense oligonucleotide, sODN), asODN treatment significantly decreased *CsWRKY17* and *CsPME6* expression in tender shoots (Fig. 6D), alongside significant reductions in PME activity and Al content in the cell wall (Fig. 6E).

It has been reported that 66 PMEs are present in *Arabidopsis* [37]. We extracted the upstream 2 kb sequences of these 66 PME genes for cis-element analysis, among which 35 genes were shown to contain W-box elements. We further extracted the amino acid sequences encoded by these 35 genes and conducted phylogenetic analysis with CsPME6, and found that AT2G43050(AtPME16) and AT3G59010(AtPME61) were clustered with CsPME6. We determined the expression levels of these two genes in WT and *CsWRKY17*-OE plants subjected to Al treatment, and found that AtPME16 expression was significantly higher in *CsWRKY17*-OE plants than in WT plants in the absence of Al. Meanwhile, under Al treatment, the expression of AtPME16 in WT and *CsWRKY17*-OE was significantly upregulated 3.0- and 4.9-fold, respectively (Fig. S10A). However, the expression of *AtPME61* showed an opposite trend compared with *AtPME16* in WT and *CsWRKY17*-OE plants (Fig. S10B). These results indicated that *CsWRKY17* overexpression in *Arabidopsis* significantly increased the expression of *AtPME16*, the homologous gene of *CsPME6*, and the expression of *AtPME16* was significantly induced by Al, which may play a positive role in the resistance of *Arabidopsis* to Al stress.

**Figure 7:**
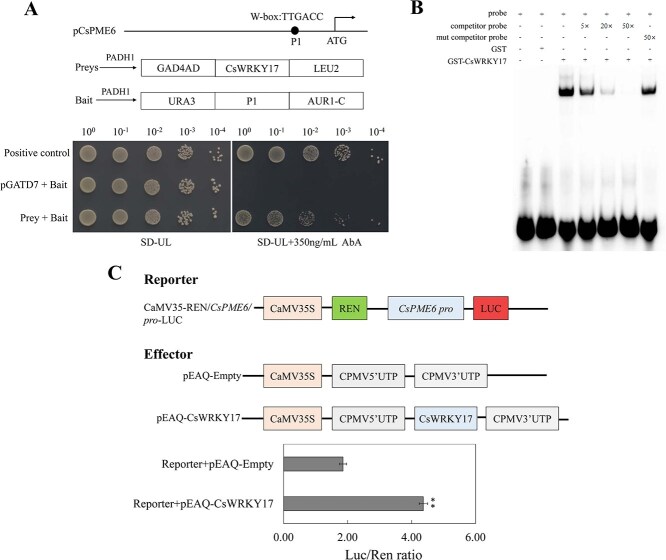
Analysis of the binding of *CsWRKY17* to the *CsPME6* promoter. (A) In Y1H assay, the AbA concentration was 350 μg/L. (B) In EMSA, the purified CsWRKY17 protein was combined with various probes; (+) represents addition, while (−) represents subtraction. (C) DLR assay implied that *CsWRKY17* regulates the expression of *CsPME6*. Asterisks denote a significant difference (^**^P < 0.01).

Additionally, to determine the potential transcriptional regulation of *CsWRKY17* on the *CsPME6* promoter, we explored the interaction between them using a yeast- one-hybrid (Y1H) assay. As shown in Fig. 7A, no basal activity of pro-*CsPME6* was identified in yeast exposed to AbA. However, upon coexpression with *CsWRKY17*, the *CsPME6* promoter induced AbA expression. This indicates that yeast cell growth was robust in the presence of AbA, suggesting that *CsWRKY17* can interact with pro-*CsPME6*. To confirm that *CsWRKY17* directly binds to the *CsPME6* promoter, we purified recombinant GST-CsWRKY17 protein and synthesized biotin probes containing W-box binding sites on pro-*CsPME6* for an electrophoretic mobility shift assay (EMSA) (Fig. 7B). EMSA revealed that the GST protein could not bind to the pro-*CsPME6* sequence. In contrast, GST-CsWRKY17 specifically bound to pro-*CsPME6*, and its binding ability was reduced by an increase in the concentration of competitor probes. Furthermore, a DLR assay was conducted to explore the regulation of *CsPME6* by *CsWRKY17* in *Nicotiana benthamiana* leaves, and the resulting change in the LUC/REN ratio was determined. The results revealed that the activity of pro-*CsPME6* was strongly promoted in the presence of *CsWRKY17* compared with that of the empty control (Fig. 7C). Taken together, these results suggest that *CsWRKY17* transcriptionally promotes *CsPME6* expression by specifically binding to the W-box element on the *CsPME6* promoter, thereby participating in Al fixation through pectin demethylesterification in the cell wall of tea plants.

## Discussion

### QTL mapping combined with transcriptomics effectively identified candidate genes for different traits in tea plants

QTL mapping has been successfully applied to key quality components such as leaf color variation and the phenological phase in tea plants [38]. However, candidate regions identified for QTL localization often remain physically distant on the genome. For annual gramineous plants like rice and wheat, QTL intervals can be narrowed down by constructing secondary isolated populations [39]. However, due to the highly heterozygous genetic background and self-incompatibility of woody plants such as tea plant, it is difficult to construct multi-population. Recent publications of high-quality chromosome-level reference sequences of the tea genome enable the effective identification of candidate genes using transcriptomic sequencing or resequencing based on annotated initial QTL localization intervals of the reference genome. For example, transcription factors such as *CsMYB75* and *CsGSTF1* [phi (F)-type glutathione transferase 1], crucial for anthocyanin accumulation, were identified through QTL mapping of an F_1_ population derived from purple and green leaf parents, coupled with comparative transcriptomic analysis of ‘Longjing 43’ and ‘Zijuan’ [36]. Similarly, an analysis of an F_1_ population from ‘Zhongcha 302’ (theacrine-free) crossed with ‘Ruyuan Kucha’ (theacrine-rich), combined with bulked segregant RNA sequencing, identified the theacrine synthase (TcS) gene as a candidate for theacrine accumulation [40]. Al tolerance in plants, a quantitative trait affected by the presence of a few genes, has been confirmed in numerous studies [41–43]. In this study, we used an artificial hybrid F_1_ population with significant differences in Al content between parents and identified the stable QTL *qAl09*. This QTL was repeatedly detected in spring, summer, and autumn in the LG09 linkage group, and contributed to 10.7% to 17.6% of the phenotype. A collinearity comparison revealed that 96.9% of the markers were found on the identical chromosome in both the genetic map and the ‘Shuchazao’ genome, confirming the high contiguity and accuracy between the genetic and physical maps [31, 44]. The *qAl09* was shown to be located in the genome on chromosome 07 in the range of 35 256 594–57 378 817, spanning ~22.1 Mb. Based on reports in the literature [32–34] and functional annotations, we further screened 41 candidate genes. By correlating gene expression levels with Al content in leaves and leaf cell walls under different Al concentrations, we identified *CsWRKY17* as a potential candidate gene associated with Al accumulation in tea plants. This gene had high correlation coefficients with Al content in leaves (*r* = 0.931) and cell walls (*r* = 0.946). In conclusion, conventional QTL mapping combined with high-throughput sequencing technology can effectively accelerate the mining of target genes that control important traits in tea plants.

### WRKY transcription factors play diverse roles in biological processes by regulating cell wall function

The plant cell walls form a natural barrier against external challenges and pathogens, as well as determining the expansion and shape of the plant cells [45]. Research has shown that WRKY transcription factors significantly affect plant growth, development, and stress responses by participating the cell wall metabolism processes [46, 47]. For example, in *Populus trichocarpa*, *PtrWRKY19* negatively regulates secondary cell wall (SCW) development in pith cells by suppressing the expression of lignin biosynthetic genes [48]. In *Fragaria vesca*, *FvWRKY48* may enhance the expression of *FvPLA* (pectate lyase), leading to modifications of homogalacturonans (HGs) in the middle lamella and tricellular junction zones. These modifications result in the degradation of pectin and softening of fruit [49]. In herbaceous peony (*Paeonia lactiflora*), *PlWRKY41a* binds to the promoter of *PlXTH4* (xyloglucan endotransglucosylase/hydrolase 4) and directly activates its expression. *PlXTH4*-overexpressing tobacco has thicker SCWs and enhanced stem strength. Additionally, WRKY transcription factors also affect Al accumulation and transport in plants. Specifically, *WRKY46* negatively regulates the malate transporter gene *ALMT1* in *Arabidopsis* by directly binding to the W-box in its promoter. A *WRKY46* mutation was shown to increase the secretion of malate and decrease Al content in root apices, thereby enhancing Al resistance in *Arabidopsis* [50]. In contrast, *OsWRKY22* has a positive regulatory effect on rice Al tolerance by promoting the expression of *OsFRDL4* and secretion of citrate [51]. Additionally, *WRKY47* has been reported to directly manipulate genes that modify the cell wall, such as *ELP (EXTENSIN-LIKE PROTEIN)* and *XTH17*, along with adjusting the Al distribution between the apoplast and symplast of roots in order to cope with Al stress [52]. In this study, the overexpression of *CsWRKY17* in *Arabidopsis* resulted in higher Al content and increased pectin demethylesterification in leaves. RNA-seq analysis revealed a 5.24-fold difference in the number of DEGs between WT plants and *CsWRKY17-*overexpressing plants under Al treatment, indicating greater sensitivity to Al in WT plants. Furthermore, reduced PME activity and lower Al content were observed in the new shoots of tea plants when *CsWRKY17* expression was silenced. These findings suggest that *CsWRKY17* enhances Al accumulation in leaves by regulating the pectin structure.

### *CsPME6* participates in Al fixation in tea leaf cell walls

Pectin, a primary constituent of the cell wall, harbors numerous negatively charged carboxylic groups that serve as the chief binding sites for metal ions [53, 54]. The pectin content was shown to correlate with Al resistance in plants, as demonstrated in studies involving maize and rice. Specifically, Al-sensitive varieties exhibited higher root tip pectin content than Al-resistant varieties, with a significant increase in pectin content following Al treatment [28, 55]. A positive correlation between Al content and pectin content in root tips was also evident in an analysis of eight local buckwheat (*Fagopyrum tataricum*) varieties [56] and rice bean (*Vigna umbellata*) [57]. Furthermore, our study showed that various Al concentrations significantly promoted total pectin content in tea plant leaves, suggesting that the level of pectin is crucial for the sequestration of Al to the cell walls tea leaves.

PMEs catalyze the demethylesterification of highly methylesterified pectin and release carboxyl groups for Al binding [19]. This process determines Al sensitivity or resistance in various plant species [55, 58, 59]. For example, HG, a major pectic polysaccharide domain in the cell wall, exhibits a disorganized distribution of methyl esterified and unesterified HG, which is linked to the inhibition of root growth caused by Al in maize [60]. Similarly, another study showed that Al-sensitive cultivars of *Pisum sativum* demonstrate lower methyl esterified pectin content in the transition zone of roots, leading to the greater accumulation of Al in the cell wall and cytosol [61]. As hyperaccumulators of Al, tea plants primarily detoxify Al by binding it to the cell wall [12]. Our findings demonstrate that Al promoted pectin methylesterase activity and deesterification in the cell wall of tea plants, which is consistent with the findings of Li *et al.* [22]. Having previously identified 66 pectin methylesterase genes [62], we found that W-box elements, WRKY protein binding sites, were present in 37 *CsPME* promoter regions. Notably, *CsPME6* was significantly upregulated under various Al concentrations, compared with the findings in the control. These correlations with *CsWRKY17* expression and variation in cell wall Al content suggests the potential role *of CsPME6* in Al fixation in the leaf cell walls of tea plants. Next, we cloned the *CsPME6* promoter and found that the W-box binding site for WRKY was present in the promoter region of *CsPME6*, allowing *CsWRKY17* interaction and regulation of gene expression. Transient knockdown of *CsWRKY17* expression in the tender shoots of tea plants significantly reduced both *CsWRKY17* and *CsPME6* expression and decreased cell wall Al content (Fig. 6). Additionally, via Y1H, EMSA, and DLR assays, we demonstrated that *CsWRKY17* could bind to the promoter of *CsPME6* (Fig. 7). This binding activity contributes to the transcriptional activation of *CsPME6*, thereby altering the pectin structure to regulate Al in the tea plant cell wall.

In summary, this study is the first to conduct the mapping of QTLs associated with Al content in the leaves of tea plants using controlled hybridized populations. The *qAl09* locus was identified within region 35 256 594–57 378 817 bp on chromosome 7. Through experiments with different Al concentrations, the cell wall was found to be the primary site for Al accumulation in both roots and leaves, with most of the Al being localized in the pectin fraction. Al increased PME activity and promoted HG DM, enhancing Al chelation in the cell wall. Comparative transcriptomic analysis identified *CsWRKY17* as a key regulator of Al accumulation in tea plants, based on the strong correlation between its expression and Al content in the cell walls of leaves. The overexpression of *CsWRKY17* in *Arabidopsis* resulted in higher Al content in leaves, increased pectin deesterification , and reduced sensitivity to Al. Additionally, the expression pattern of *CsPME6* was highly consistent with that of *CsWRKY17* under various Al concentrations. Cloning and sequence analysis of the *CsPME6* promoter revealed the existence of a WRKY transcription factor binding site (W-box). Further experiments demonstrated that *CsWRKY17* binds to the *CsPME6* promoter and positively regulates Al enrichment in tea plants by directly activating *CsPME6* expression*.* Therefore, this study provides new insights into the molecular mechanisms and regulatory networks underlying Al enrichment in the leaf cell walls of tea plants.

## Materials and methods

### Plant materials and experimental design

An F_1_ population was obtained via controlled hybridization of *C. sinensis* var. *sinensis* ‘Yingshuang’ (‘YS’) and *C. sinensis* var. *pubilimba* ‘Beiyao Danzhu’ (‘BD’) through pseudo-testcross. A genetic map was constructed using 406 simple repeat sequences (SSRs) and 6042 SNP markers to genotype 137 individuals, as detailed in previous studies [63].

One-year-old plug seedlings of ‘E’cha 1’ were rinsed with deionized water and transplanted into nutrient solutions, as described in a previous report [25]. The plants were hydroponically cultured in a greenhouse under a 16/8-h day/night cycle, with relative humidity being maintained at 65%–85% and temperature at 22–25°C. The solutions were adjusted to pH 5.0 with 1 mM NaOH or H_2_SO_4_, and refreshed every 7 days until the first trifoliate leaves appeared. Treatments with five different Al concentrations (0, 0.25, 0.5, 1, and 2 mM) were established, with each treatment being performed in triplicate. After 30 days, the third leaves from each treatment were harvested, frozen in liquid nitrogen, and kept at −80°C.

### Al content determination

Al content was detected the dry ash analysis. A 0.1-g sample of powder was heated in a muffle furnace at 550°C for 3 h and left overnight before the furnace door was slightly opened. Samples were removed once the internal temperature reached 30°C. Next, 25 ml of HCl (1:1, concentrated hydrochloric acid/water) was added to each crucible to dissolve the sample, which was then filtered through a 0.45-μm inorganic membrane into a 10-ml centrifuge tube for measurement using an inductively coupled plasma optical emission spectrometer.

### Pectin and hemicellulose content determination

The measurement of pectin in tea leaves and roots was performed using the *m*-hydroxybiphenyl method [64], with GalA as a standard. Hemicellulose content was determined by using the 3,5-dinitrosalicylic acid method with a Hemicellulose Content Assay Kit (Boxbio, Beijing, China).

### Cell wall extraction, PME activity, and DM measurements

The cell walls of tea leaves and roots were extracted using an alcohol-insoluble solid method as described by Luo *et al.* [65]. Cell wall fractionation was performed as follows [66]. First, 500-mg cell wall samples were weighed and mixed with 40 ml of 50 mM Na_2_CO_3_(with 20 mM trans-1,2-cyclohexanediaminetetraacetic acid), followed by shaking at 25°C and 150 rpm for 24 h. Then, the mixture was centrifuged and the obtained supernatant was collected. The precipitate was rinsed with 10 ml of extractant again, and the supernatants were then combined to obtain pectin components. The residue was subsequently added to 4 M KOH (including 20 mM NaBH₄) to extract the hemicellulose fraction using the same procedure, and the pH was adjusted to 6.0 using glacial acetic acid. The remaining residue was considered to be cellulose. The three cell wall fractions were freeze dried for the analysis of Al content, which was calculated according to the weight of the cell wall. To determine PME enzyme activity, 1 g of sample was quickly ground into powder with liquid nitrogen, homogenized with 9 ml of PBS buffer (pH = 7.2–7.4, 0.01 M), and centrifuged at 4°C and 12 000*g* for 15 min. Finally, the supernatant was used to analyze PME activity using an ELISA kit, in accordance with manufacturer’s instructions (Meibiao, Jiangsu, China). To determine the DM, 0.1 g of tissue was homogenized with 1 ml of 80% ethanol, followed by centrifugation at 8000 rpm and 4°C for 10 min. The residue was retained and the supernatant was discarded. The above steps were then repeated. Next, 1 ml of extraction liquid was added to the precipitate, mixed thoroughly, and boiled for 1 h. The mixture was then cooled in running water and centrifuged at 8000 rpm and 4°C for 10 min. The supernatant was analyzed using a DM Assay Kit following the manufacturer’s instructions (Comin, Jiangsu, China).

### FT-IR analysis

To analyze FT-IR spectra of the cell wall, a Nicolet iS 50 spectrometer (Thermo Fisher, Waltham, MA, USA) was used. First, 2 mg of cell wall and 200 mg of pure KBr were mixed and ground evenly, after which the mixture was placed in a mold and pressed into a transparent sheet using an oil press for infrared spectrometric analysis. Each sample was recorded at 4 cm^−1^ intervals within the spectral range of 4000–400 cm^−1^ over 32 scans.

### Pectin immunofluorescence experiments

For the observation of pectin immunofluorescence, all samples were exposed to formalin-aceto-alcohol (FAA) solution (100 ml; consisting of 90 ml of 50% ethanol, 5 ml of acetic acid, and 5 ml of formaldehyde), fixed for 24 h, paraffin embedded, sliced into sections with a thickness of 4 μm, dewaxed, and rehydrated. The localization and distribution of pectin were detected by immunohistochemistry as follows. First, the tissue sections were placed in antigen repair fluid (0.01 M citrate sodium buffer, pH 6.0) for antigen repair. After lightly drying, a circle was drawn around the sections using a histochemical pen to block the flow of antibodies. Then, an autofluorescence quenching agent was added to the circle for 5 min, followed by rinsing with water for 10 min and incubation in 3% bovine serum albumin (BSA) for 30 min. Next, the tissue sections were incubated with the primary antibody (LM19 and LM20; Plantprobes) overnight at 4°C and then with the secondary antibody [Alexa Fluor 488 Anti-Rat IgG (H + L), Invitrogen, A-11006] for 50 min at room temperature. Finally, the tissues were observed using a fluorescence microscope (Eclipse C1, Nikon, Tokyo, Japan). All images were taken at the same magnification for three biological replicates. The fluorescence intensity was measured using ImageJ by calculating the mean intensity value.

### RNA-seq and gene expression analysis using qRT-PCR

Total RNA was extracted from tea leaves treated with 0, 0.25, and 1 mM for transcriptomic sequencing. The cDNA libraries were analyzed using an Illumina Novaseq platform (Metware, Wuhan, China). For each sample, high-quality clean reads were mapped to the reference genome [67] using Hisat2. The gene expression levels were standardized using fragments per kilobase per million reads values. DEGs, significantly enriched GO terms, and KEGG enrichment were analyzed as described previously [25]. qRT-PCR and relative gene expression analyses were also conducted as described previously [62]. Related primers are presented in Table S8.

### Subcellular localization of *CsWRKY17*

The primers *CsWRKY17*-F and *CsWRKY17*-R were designed based on the *CsWRKY17* sequence in the genome (Table S8) to amplify its full-length CDS. The CDS was attached to the PC1300s-GFP vector via homologous cloning. The recombinant plasmid PC1300S-CsWRKY17-GFP (35S::CsWRKY17::GFP) and the empty vector PC1300S-GFP (35S::GFP) were transformed into *Arabidopsis* protoplasts using a nuclear marker. After incubation in the dark at 28°C for 8–10 h, the subcellular localization of *CsWRKY17* was observed using a confocal laser microscope (FV1200, Olympus, Tokyo, Japan).

### Stable transformation experiment of *CsWRKY17*

For the experiment on the stable transformation of *CsWRKY17*, the CDS of *CsWRKY17* was amplified by PCR and cloned into the pMD19T vector. The product was recombined into a pCAMBIA1301 vector containing the 35S promoter using the Gateway (Invitrogen). The constructed overexpression vector was transformed into *Escherichia coli* recipient cells, and positive clones were confirmed by sequencing. The recombinant plasmid was electrically inserted into *Agrobacterium tumefaciens* GV3101 to infect *Arabidopsis* Col-0 using the standard flower dipping method [68]. Transgene-positive plants were identified on 1/2 MS medium containing 35 mg·L^−1^ hygromycin, and specific primers were used for PCR qualification until stable transgenic T3 homozygous lines were obtained.

*Arabidopsis* seedlings grown on 1/2 MS solid medium for 2 weeks were selected to analyze the effects of Al treatment on *Arabidopsis* leaves. The procedure was as follows: 2 ml of CK solution or treatment solution was added to each well of a 24-well plate, and seedlings were carefully transferred, one per well, ensuring that roots were immersed in the treatment solution. All seedling transfers were performed in a super-clean workbench to prevent bacterial contamination. The 24-well plates were cultured in an artificial climate chamber (light/dark period, 16/8 h; temperature, 22°C; relative humidity, 70%) for 2 days, after which leaves were collected for further analysis.

### *CsWRKY17* suppression analysis in tea shoots

AsODNs targeting *CsWRKY17* were designed using Soligo software (http://sfold.wadsworth.org/cgi-bin/index.pl). ‘One and a bud’ tender shoots from ‘E’cha 1’ were harvested and treated with a 20 μM asODN solution for 48 h, and sODNs served as controls. Samples were collected to analyze cell wall Al content, PME activity, and related gene expression.

### Cloning of the *CsPME6* promoter

To clone the *CsPME6* promoter, specific primers (Table S8) were designed based on the upstream promoter sequence of *CsPME6* obtained from the ‘Shuchazao’ genome sequence. The genomic DNA of *CsPME6* from ‘E’cha 1’ was extracted using a Plant Genomic DNA Kit (TianGen, Beijing, China) following the manufacturer’s instructions. The full-length amplified sequences were cloned into the pUC57 simple TA vector and confirmed by sequencing. Subsequently, the cis-acting regulatory elements of the *CsPME6* promoter were predicted using the plantCARE database (http://bioinformatics.psb.ugent.be/webtools/plantcare/html/).

### Y1H, EMSA, and dual-luciferase assay

The yeast one-hybrid (Y1H) assay was performed in accordance with established protocols [69]. Specifically, the *CsPME6* promoter fragment was cloned into the pAbAi vector, and *CsWRKY17* was integrated into the pGADT7 vector. The positive controls included pGADT7-53 and p53-pAbAi, while pGADT7-T and p53-pAbAi served as negative controls.

Meanwhile, the interaction between the *CsWRKY17* protein and the *CsPME6* promoter was evaluated using EMSA. For EMSA, the ORF of *CsWRKY17* was cloned into the pGEX-6p-1 vector, which incorporates a glutathione-*S*-transferase (GST) tag. The GST-fused protein was purified using standard methods [70]. The EMSA experiments followed the protocol described by Li *et al.* [71].

In the dual-luciferase assay, full-length *CsWRKY17* was cloned into the pEAQ vector as the effector plasmid, whereas the *CsPME6* promoter was subcloned into the pGreenII 0800-LUC vector as the reporter plasmid. The effector and reporter plasmids were mixed at a 9:1 ratio and injected into tobacco leaves. After 3 days of cultivation under suitable conditions, luciferase activity was measured using dual-luciferase assay reagents from Vazyme (Nanjing, China).

### Statistical analysis

For Al content in the F_1_ population, the mean, SD, CV, kurtosis, and skewness were calculated using GraphPad Prism 7.0. QTL detection was performed using MapQTL 6 with interval mapping and restricted multiple QTL model (rMQM) methods, as described previously [63]. Gene expression profiles were visualized using heat maps generated with TBtools [72]. The homologous sequences of *CsWRKY17* from different species were retrieved from NCBI using blastp with the CsWRKY17 protein sequence as a template. Multiple sequence alignment was performed using DNAMAN 6.0. The phylogenetic tree of WRKY sequences was constructed by the neighbor-joining method in MEGA 5.0 software. Differences among treatments were analyzed using LSD test at a significance level of 0.05, one-way ANOVA, using GraphPad Prism 7.0.

## Supplementary Material

Web_Material_uhaf085

## Data Availability

The raw RNA-seq data are available in the NCBI Sequence Read Archive under BioProject PRJNA1137106 and PRJNA1229406.
